# Development and Optimization of a New UPLC-UV/MS Method through DoE and MLR for Detecting Substandard Drug Products to Treat Tuberculosis

**DOI:** 10.3390/molecules27207141

**Published:** 2022-10-21

**Authors:** Javier Suárez-González, Amor R. Cáceres-Pérez, Alexis Oliva, Ana Santoveña-Estévez, José B. Fariña

**Affiliations:** 1 Departamento de Ingeniería Química y Tecnología Farmacéutica, Facultad de Farmacia, Universidad de La Laguna, 38200 San Cristóbal de La Laguna, Spain; 2 Instituto Universitario de Enfermedades Tropicales y Salud Pública de Canarias, Universidad de La Laguna, 38200 San Cristóbal de La Laguna, Spain; 3 Programa de Doctorado Ciencias Médicas y Farmacéuticas, Desarrollo y Calidad de Vida, Universidad de La Laguna, 38200 San Cristóbal de La Laguna, Spain

**Keywords:** tuberculosis, design of experiment, multi-linear regression, central composite design, quality by design

## Abstract

Drug products used for treating tuberculosis are one of the most widely reported medicines to be classified as falsified or substandard in low- and middle-income countries, representing a major hazard to health. The aim of this study was, firstly, to develop an ultra-performance liquid chromatography (UPLC) method which is able to analyze fixed combination tablets with up to four active pharmaceutical ingredients, including isoniazid, pyrazinamide, rifampicin, and ethambutol. Secondly, we aimed to optimize it through the design of experiments and multi-linear regression based on a central composite design and to validate it according to the guidelines of the International Conference on Harmonization. The application of this tools enabled the identification of the influential factors (flow rate, formic acid, and temperature) and their effects on the studied responses (retention factor and percentage for each drug) as part of the quality by design approach. The method proved to be to be linear in the range from 5.0 to 15 µg/mL for isoniazid, pyrazinamide, and rifampicin, being precise (<1%) and accurate (97–101%). In addition, the method validated for ethambutol proved to be linear from 1.4 to 4.2 µg/mL, as well as precise (0.54%) and accurate (97.3%). The method was stability indicated for all the active pharmaceutical ingredients studied and was able to detect two substandard formulations sampled on the African market.

## 1. Introduction

Every year, millions of people die because of the presence of falsified and substandard medicines on the pharmaceutical market. The World Health Organization (WHO) defines falsified medical products as those which are deliberately fraudulent, misrepresenting their identity, composition, or source, and it defines substandard medical products as those which are authorized drug products but fail to meet either their quality standards and/or their specifications [1,2]. In this latter case, the quality of the drug product can be affected from manufacture to storage, as well as during delivery. This is particularly important in countries in climate zones of type IV, where the temperature and relative humidity (RH) are high [3].

The presence of poor-quality products is difficult to quantify, but there is no doubt about the consequences of their presence in the pharmaceutical market. These include, for example, the endangerment of health, the prolongation of illness (even death), the promotion of antimicrobial resistance, the spread of drug-resistant infections, and the creation of distrust regarding the effectiveness of vaccines and medicines. All this leads to a lack of confidence in health professionals and health systems [2].

WHO estimates the observed failure rates of substandard and falsified medical products in low- and middle-income countries to be 10.5%. However, it is difficult to know the current absolute number of substandard and falsified drug products. This is the reason why WHO implemented a Global Surveillance and Monitoring System (GSMS) in order to locate and report the presence of these products. In this global system, the African region accounted for 42% of the global reports, even though not all the countries have access to a certified laboratory which meets the conditions for the analysis and detection of substandard and falsified medicines. Antibiotics and antimalarial drugs were the most widely reported medicines from 2013 to 2017, accounting for 16.9% and 19.6%, respectively. In the third position were anesthetics, painkillers, and lifestyle products (products for cosmetic use, erectile dysfunction, bodybuilding, and dieting), accounting for 8.5% [1,2,4].

Furthermore, there are other platforms, such as the infectious diseases data observatory (IDDO), which developed a drug quality map based on data from different studies published since 1985. This database reflects the conclusions of the GSMS concerning the most frequently falsified/substandard medicines detected, as well as the region with the most reports in this sense [5].

As mentioned previously, the presence of falsified and substandard medicines constitutes a major hazard with respect to prevalent diseases, such as tuberculosis (TB). This situation represents a high risk for TB patients due to the complexity of their treatment and the well-known increasing level of resistance [6,7,8,9,10]. TB is treated with at least four active pharmaceutical ingredients (APIs), including rifampicin (RFP), isoniazid (INH), pyrazinamide (PZA), and ethambutol (EMB), for 6 months or even more [11,12].

Due to the importance of the detection of substandard and falsified medicines, a research project called ISACAM (in Spanish, Instauración de un Sistema para el Aseguramiento de la Calidad de Medicamentos utilizados en el tratamiento del SIDA y enfermedades tropicales descuidadas) was initiated, with the primary aim of establishing a quality assurance system for drug products used to treat TB, malaria, and HIV/ADIS in the Islamic Republic of Mauritania, as there are no reports on the quality of drug products in this market [13].

In order to detect the presence of falsified and substandard medicines, it is essential to use an appropriate analytical method in order to analyze fixed-dose combinations tablets (FCT) with up to four different APIs, each one with its own chemical characteristics. The *United States Pharmacopoeia* (USP) recommends the analysis of an FCT of EMB, INH, PZA, and RFP, as well as the use of different mobile phases, detectors, and columns with different specifications in terms of the dimensions, particle size, and packing (the L1 column is used for INH, PZA, and RFP and L10 is used for EMB) [14]. This procedure requires the preparation of multiple samples and mobile phases, as well as the acquisition of different columns, which increases the time required to analyze all the APIs and could represent a problem due to the instability of some APIs, such as RFP or INH [15,16,17,18]. Other authors have used similar methods, but all of these have in common the use of different columns, long run times, and complex sample preparation. Moreover, in some cases, EMB has not been analyzed due to its poor absorption in the ultraviolet-visible range [17,19,20,21,22].

Hence, a new method for the analysis of the FCT of four APIs is necessary in order to analyze the quality of the anti-TB medicines available on the market. This method should be developed and optimized using the principles of quality by design (QbD), replacing old variables one at a time (OVAT). This approach makes it possible to study interactive effects between variables through the use of the design of experiments (DoE) and multiple linear regression (MLR) [23,24,25]. DoE helps to establish a cause-and-effect relationship between the factors and responses, thus reducing the time, effort, and resources required. The key principles of DoE are replication, randomization, and error control through the use of several designs, such as Taguchi, Placket–Burman, the central composite design, Box–Behnken design, etc. [26,27,28,29,30,31,32,33]

The aim of this study was to develop, optimize, and validate an ultra-performance liquid chromatography (UPLC) method to quantify APIs used as the first-line therapy for treating TB, using DoE and MLR with a central composite design. Furthermore, the aim was to evaluate the capability of the method for substandard detection and quality control. This new method could enable the detection of substandard drug products representing anti-TB combinations from low-income countries.

## 2. Results and Discussion

### 2.1. Optimization of the UPLC Method

The optimization process was performed using a central composite design. A total of 14 experiments and 6 replicates of the central point were analyzed. For this purpose, three factors (column temperature (T), flow rate (F), and percentage of formic acid in the mobile phase, FA) were analyzed. The variation range for each factor was previously fixed based on the OVAT methodology (i.e., one parameter was changed, while the others were kept at a constant level) [23,34].

In this study, two responses were used as critical quality attributes (CQAs) to determine the quality of the analytical method: (1) the retention factor (k’), which allows for the identification of the drug, and (2) the nominal amounts of INH, PZA, and RFP, expressed as percentages used for their quantification (%API) [35].

The factors and responses for each run are shown in Table 1.

In the case of INH, the results based on ANOVA for both responses indicate that the model proposed is appropriate, since the lack of fit was not significant and, therefore, the total error was used to determine which model term was significant [36]. For the k’ response, only the FA as the main factor and FA as the quadratic term (FA × FA) were significant (*p* < 0.05), although with different effects. Thus, 14.7% was the main term value, whereas a decrease of 6.5% was observed for the quadratic term (Table 2) [37] The results of the canonical analysis show that the stationary point of the fitted surface is considerably distanced from the work zone, especially for the T and FA factors (T = 35.03 °C; F = 0.400 mL/min; FA = 0.0136%). In addition, it is a saddle point, since the eigenvalues obtained are of mixed signs.

For the %INH response, all the second-order factors were significant (*p* < 0.05), particularly F, with an effect of 3.85%, compared to an average value of 2.0% for the other two factors. With regard to the main terms, only the F showed a significant effect, which was approximately six-fold greater compared to the quadratic term average. In this case, the results of the canonical analysis indicate that the stationary point was close to the work zone (T = 31.3 °C; F = 0.412mL/min; FA = 0.0093%), although it was also a point saddle.

However, it is necessary to verify that this situation does not lead to an undesirable response. For the %INH response, a predicted value of 106.3% was obtained, whereas for k’, it was 1.08, both values being very close to nominal. Under these circumstances, it is considered unnecessary to modify the central point conditions. To confirm this, the method was optimized using the lexicographic approach [37]. In such a situation, an ordered minimization process is applied [37], where each response has an assigned level of priority. The highest priority response variable is the analytical method uncertainty, whereas the quality of the peak separation and integration, i.e., k’, is used as the lowest priority response variable. In both cases, a maximum difference must be established from the target values to maintain the quality of the analytical method, in accordance with the compromise support problem (cDSP) strategy, where the deviation function is minimized. Oliva et al. [27,28] applied this methodology during the validation process of analytical methods used in pharmaceutical analysis.

For the primary response, the deviation function used was a sample estimated from the squared difference between the measurements and the true value [yi − 100]^2^, whereas for the secondary response, we proposed [k’ − k_goal_’ ]^2^, with an upper bound of 0.10 [27].

**Figure 1:**
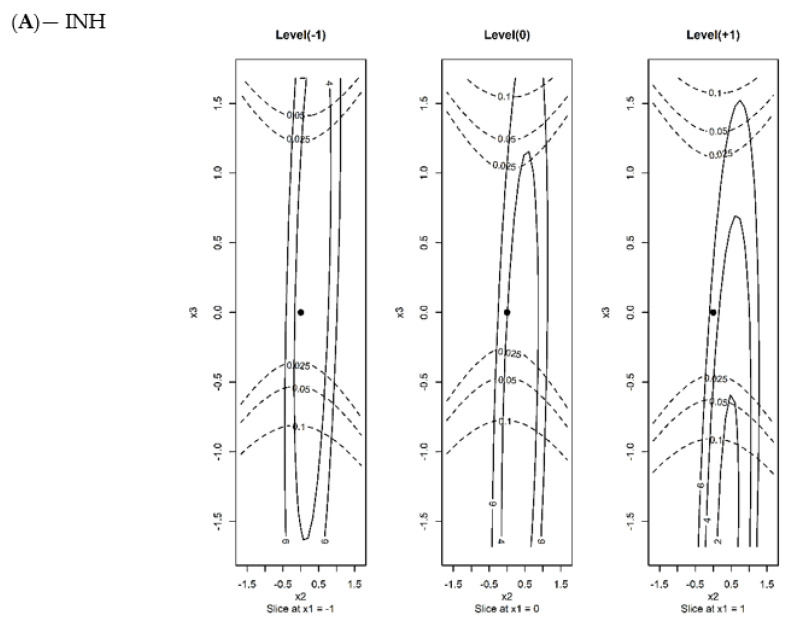

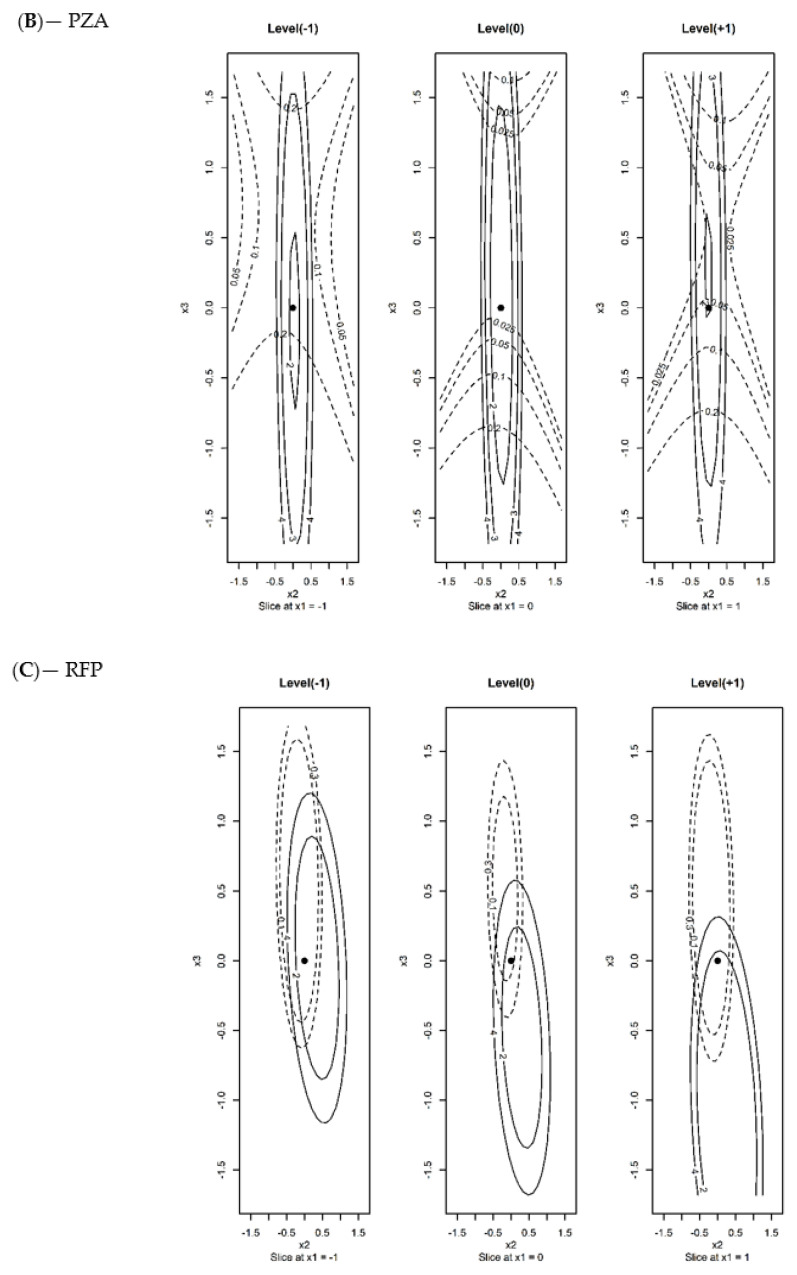
The 2D contour levels for the primary difference function [yi − 100]^2^ (continuous line and secondary difference function [k’ − k’_goal_]^2^ (dotted line) for the variables of the column temperature (X_1_), flow rate (X_2_), and formic acid content (X_3_), corresponding to the INH (**A**), PZA (**B**), and RFP (**C**) drugs. The black points show the nominal conditions. In all situations, the level of variability for the % drug responses was around 2%, although this level was twice as large for INH, and a high content of formic acid was observed.

Figure 1 depicts the contour levels of the plane for the variables FA and F for the three levels of the column temperature variable T (−1, 0 and +1) for the primary response, [yi − 100]^2^ (continuous line), and for the secondary response, [k’ − k_goal_’ ]^2^ (dotted line), for the three compounds. The central point of the experimental design was also included. Only two levels of the curves for the primary response were graphically shown, being 2.0% and 4.0%, except for %INH, where the 4% and 6% values were used. The results obtained indicate that the nominal conditions (i.e., the central point of the experimental design) fulfill the validation requirements. Moreover, the level of variation in the experimental conditions is relatively high, and both responses remain below acceptable values, even for a high level of FA. In the case of the secondary variable, the upper bound could decrease to 0.025 under certain conditions (see Figure 1A). This demonstrates the robustness of the proposed analytical method. The same strategy was applied for the other two APIs analyzed. The results obtained for both APIs and responses are summarized in Table 2.

In the case of PZA, we found that, for the k’ response, the quadratic term (FA × FA) contributed significantly to the model, whereas T, as main factor, was more influential (≈6.0%). For the %PZA drug response, only the main term F was significant, but it showed a greater effect (12.8%), being similar to those observed for INH.

Regarding the RFP, the main terms F and FA contribute significantly to the model in terms of the %API response, although its effect was different, being 4.5 times higher for the term F, whereas the F and FA under the quadratic terms were found to be only slightly influential (3.5% on average). A similar situation was observed for the k’ response, where F as main term and F as the quadratic term (FA × FA) contributed significantly to the model, and the effect of the main term was higher than the quadratic term (8.96% vs. 2.69%).

In all these situations, the eigenvalues were of mixed signs, indicating that it was a saddle point, whereas the canonical analysis showed that the stationary point of the fitted surface was fairly distanced from the experimental zone, especially in the case of the k’ response for RFP, which was outside the studied region (data not shown).

In the process of the robustness evaluation, the INH was appropriately resolved. The values of k’ varied between 1.13 and 1.22, whereas the % drug response varied between 102.1% and 106.8% when the variations in the formic acid content of the mobile phase, flow rate, and column temperature were found to be within the tolerance margin. This corresponds to twice the standard deviation of repeatability for the center point, in accordance with the criteria proposed by Destandau et al. (2006) and Le Mapihan et at. (2004) [37,39]. In such a situation, the level of variability was 2.1% for %API and 3.94% for the k’ responses, respectively. A similar situation was observed for the other two drugs analyzed, although the level of variability was slightly higher for the %API response, being 2.37% and 2.84% for RFP and PZA, respectively, whereas the API content varied from 97.5–103.0% for PZA and 98.8–103.6% for RFP.

### 2.2. Method Validation

Taking into account the fact that the conditions used for the center point showed good results in terms of their variability, the following conditions for T, F, and FA were selected for the method validation: 30 °C, 0.4 mL/min, and 0.01% of FA. 

The results of the method validation are shown in Table 3. 

**Figure 2:**
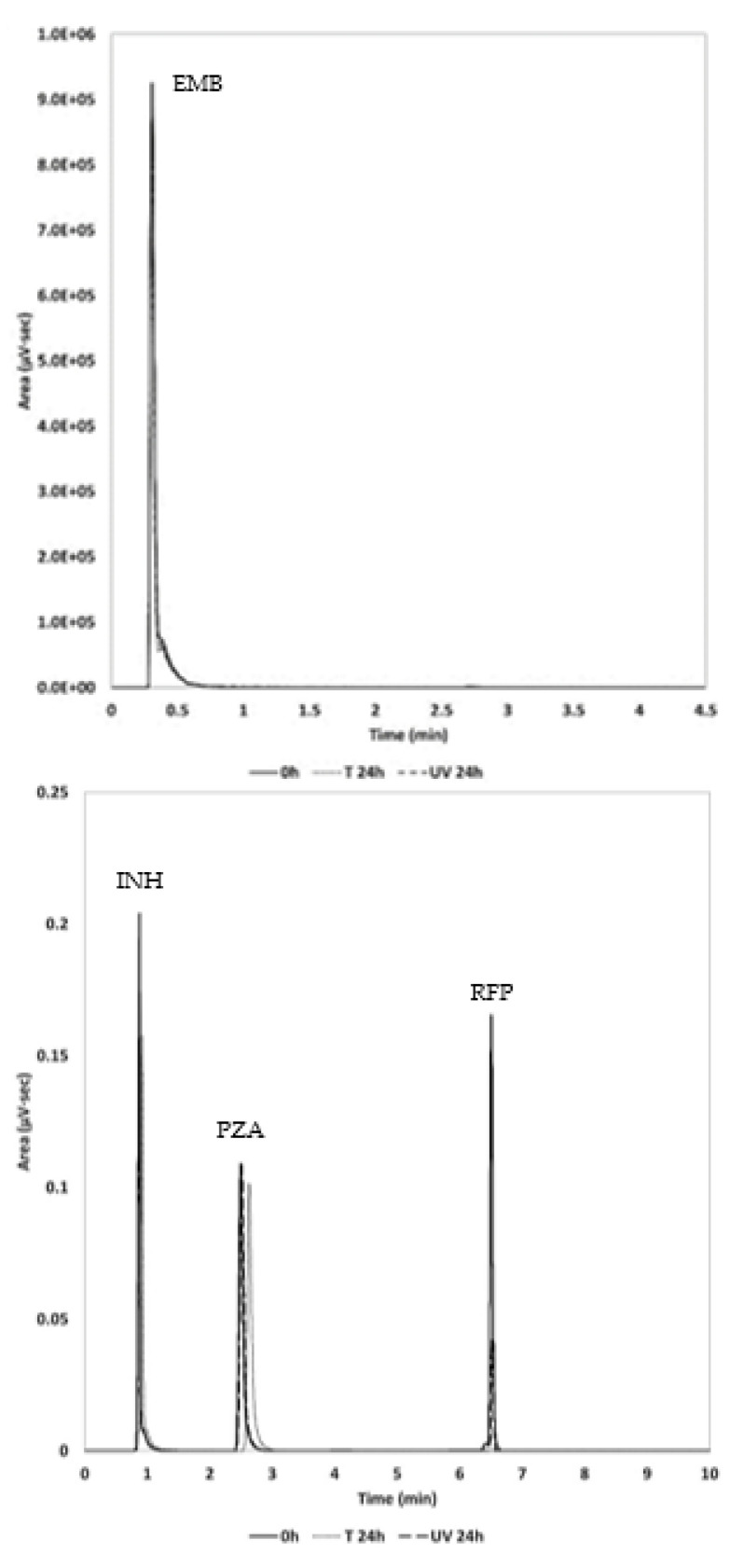
Chromatogram obtained from the analysis of a standard solution with 2.8 µg/mL of EMB and 10 µg/mL of INH, PZA, and RFP.

The ANOVA confirmed the linearity of the method for each API through the rejection of the null hypothesis of deviation from linearity for a significant level of 0.05. In addition, the methods showed coefficients of variation below 5% for all the APIs, as well as values of accuracy and precision of 97–103% and <1%, respectively. In addition, the detection and quantification limits were low enough to evaluate the quantity of APIs in the marketed drugs. In Figure 2, a chromatogram obtained from a standard solution, which combines all APIs, is shown, which is used for UPLC-UV/mass spectrometry (MS) method.

All APIs were analyzed by UV and MS. However, the results for the validation of MS signals for INH, PZA, and RFP showed a coefficient of variation and quantification limit higher than 10% and 10 µg/mL (data not shown). This might be due to the high concentration used in UV in order to obtain an acceptable signal-to-noise ratio in comparison, for example, with EMB. However, it must be highlighted that, for all the APIs analyzed by MS, the precision and accuracy were below 1.5% and greater than 97%, respectively.

This developed method is very versatile, as it enables the analysis of three or four APIs by simply adding a second detector to the system in the case of the presence of EMB. In addition, due to the higher sensitivity of the MS detector, it is easier to detect degradation products that may be toxic during its period of use, even when they are not detected by a UV detector.

The results for the pure standards of anti-TB drug storage under stress conditions are shown in Table 4. As can be seen, a degradation of all the APIs was detected for both conditions, the temperature and light. This degradation is more predominant in RFP than in the rest of APIs, being 0% at 2 h of storage at 80 °C and 24% at 4 h of storage under light. In the case of the remaining APIs, a degradation greater than 10% was detected, except in the case of PZA, which was the most stable API. In addition, no degradation product was detected for any API under our analytical conditions.

According to these results, this method is a stability-indicating method, as it detects the degradation of the API, which is important for the quality control of anti-TB drug products. This new method is quicker, easier, cost-saving, and versatile compared to the method recommended by USP, as it only requires one column and one mobile phase, and all APIs are analyzed in less than 10 min. The USP method requires a complete change of the whole chromatographic system in order to analyze it. Derivatization procedures have been proposed for the analysis of drugs with poor molar extinction coefficients, such as EMB. However, this procedure is not suitable for the analysis of commercially available drug products due to its complex matrix. In addition, when this procedure was followed, a poor K´ value was found for the derived EMB and, for example, the RFP [21]. Other liquid chromatographic methods used for the analysis of anti-TB based on MS have been reported, but these methods simply use this detector. In addition, they are not developed for the quantification of combination samples, and unknown peaks have been detected in pure standard chromatograms [19,40,41]. However, the new proposed method uses MS as a supplementary detector for the determination of EMB, as the rest of the APIs showed an optimum separation with the UV detector due to the method optimization that was previously performed. Furthermore, this method is the only stability-indicating method for the analysis of the EMB, INH, PZA, and RFP which has been developed and optimized using the DoE and MLR with a central composite design and can be used in either low- and middle-income countries, as well as the rest of the world.

In addition, this method could be defined as a method of choice for all applications following the RGB additive color model. The score obtained using this model was 88.6, which means that all attributes evaluated obtained a satisfactory result [42]. This is related to the relatively low toxicity of the solvents used, except for formic acid, and the low volume of mobile phase required for one run using the ultra-performance liquid chromatography (UPLC) system. In addition, four APIs were analyzed in a single injection, which took less than 10 min, thus saving time and money.

### 2.3. Quality Control Application

**Figure 3:**
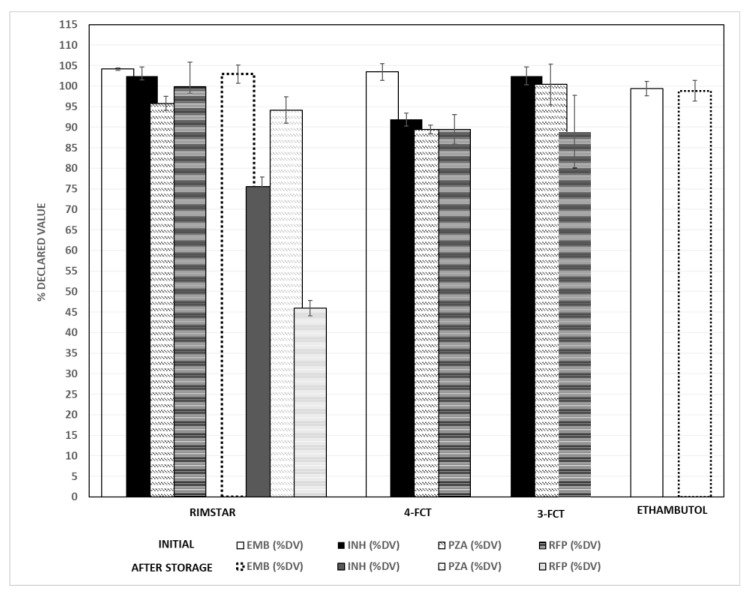
Percentage of API according to the declared content for each fixed-dose combination tablet (FCT) analyzed. DV: declared value, EMB: ethambutol, INH: isoniazid, PZA: pyrazinamide, RFP: rifampicin.

The results for the quality control of drug products sampled from Spain and the Islamic Republic of Mauritania are shown in Figure 3. All APIs based on the Rimstar^®^ formulation (a combination of EMB, INH, PZA and RFP commercialized in Spain) were between the 95 and 105% values, expressed as the declared value (DV). In the case of 4-FCT combination (EMB, INH, PZA, and RFP), from the Islamic Republic of Mauritania, only EMB was inside these limits. In the case of the triple combination (3-FCT, INH, PZA, and RFP), RFP showed a low %DV, being 88.9 ± 8.8%. Thus, this drug product could be categorized as substandard and was not placed under accelerated conditions. Tablets with EMB alone were also analyzed, showing 100% of the DV. After six months of storage in a climatic chamber at 40 ± 2 °C and 75 ± 5% of RH, the percentage of EMB and PZA remained unchanged. However, in the drug product Rimstar^®^, the content of INH and RFP was noticeably reduced.

Our results agree with the previously published results of other authors, although the variability in the degradation of the APIs is considerable [15,17,18,43,44]. The method was proven to be capable of analyzing FCT tablets of multiple APIs, as the drug products used as a quality reference (Rimstar^®^) showed API contents between the limits.

## 3. Materials and Methods

### 3.1. Materials

INH was generously donated by Acofarma^®^ (Barcelona, Spain). RFP was purchased from Fagron^®^ (Barcelona, Spain). EMB and PZA were purchased from Sigma-Aldrich^®^ (Madrid, Spain). Acetonitrile and formic acid were of a mass spectrometry grade (Sigma-Aldrich^®^, Madrid, Spain), and purified water was obtained from a water purification system with a conductivity of 0.055 µS/cm at 25 °C (Type 1 Water Purification System, ThermoFisher Scientific, Madrid, Spain). Methanol reactive category was used to sample the treatment (Sigma-Aldrich^®^, Madrid, Spain).

### 3.2. UPLC Analysis

Analytical separations were formed in an Acquity UPLC^®^ H-Class System consisting of a quaternary solvent pump, a sample manager flow through a needle unit, which was responsible for the injection of the sample, an XSelect^TM^ CSH^TM^ C18 (75 mm × 2.1 mm id, 2.5 µm) reversed phase column, and two different detectors, all obtained from Waters (Milford, USA). The mobile phase consisted of acetonitrile and a mixture of water with a known percentage of formic acid, called solvents A and B, respectively. The analysis was performed following a gradient method at a flow rate of 0.4 mL/min with an initial step of 100% B for 2.5 min, as shown in Table 5.

In order to analyze all the APIs, two detectors were used. On the one hand, a wavelength of 254 nm was set using a Acquity tunable UV detector (Waters, Milford, MA, USA). On the other hand, an electrospray ionization in the positive and negative ion mode with a capillary cone of 0.8 kV was used for an Acquity triple quadrupole MS instrument (Waters, Milford, USA). The source and desolvation temperatures were 600 °C. Additionally, nitrogen was used as a desolvation gas. A selected ion recording (SIR) was established for the EMB (116.00 Da) at a cone voltage of 30 V. In addition, an MS scan was also performed. The injection volume was 10 µL. The control of the chromatographic system, as well as the collection and processing of data, were performed using the software EmpowerTM (Waters, Milford, CT, USA).

### 3.3. Design of Experiments

A design of experiment (DoE) approach was used to evaluate the effects of three factors (T, F, and FA) through the response surface under the nominal conditions. The following equation was applied:

(1) $$y=\beta_{0}+\overset{3}{\underset{i=1}{\sum }}\beta_{i}x_{i}+\overset{3}{\underset{i<1}{\sum }}\overset{3}{\underset{j=2}{\sum }}\beta_{\mathrm{ij}}x_{i}x_{j}+\overset{3}{\underset{i=1}{\sum }}\beta_{\mathrm{ii}}x_{i}^{2}$$

where y is the response, β_0_ is the intercept, β_i_ refers to the main coefficients, β_ij_ refers to the two-factor interaction coefficients, and β_ii_ refers to the quadratic coefficients. 

One standard solution was elaborated for each run, with concentrations of 2.8 µg/mL for EMB and 10 µg/mL for INH, PZA, and RFP, in the method optimization and robustness study, with mobile phase as the diluent.

The R-program [45] was used for the design and data analysis. The lack-of-fit test was used to determine whether the model proposed was suitable for the data interpretation. If the result of this test was not significant, the total error was used to evaluate which the model coefficients were significant through Student’s *t*-test. The method proposed by Destandau et al. (2006) was applied [38] to quantify the relative effect of each factor on the response (% effect).

### 3.4. Analytical Method Validation

The method was evaluated according to the International Council for Harmonization of Technical Requirements for Pharmaceuticals for Human Use (ICH) guidelines, through the evaluation of the specificity, linearity, accuracy, and precision [46].

Standard solutions of EMB with concentrations ranging from 1.4 to 4.2 µg/mL and 5.0 to 15.0 µg/mL for INH, PZA, and RFP were used as standards for the analytical method validation (n = 30), with mobile phase as the diluent.

The linearity of the method was confirmed by the ANOVA of linear regression through the rejection of the null hypothesis of lack-of-fit (α = 0.05). The accuracy was evaluated by the analysis of nine standard solutions of three different concentrations over the specified validation range. The average percentage recoveries should be between 97% and 103% in order to comply with the requirements. The system precision, expressed as the repeatability, was determined from six replicate injections of a sample, and the coefficient of variation should be less than 1%. Moreover, detection and quantification limits were calculated for each API based on the signal-to-noise approach.

During the study, system performance was controlled by parameters such as the area, retention time, tailing factors, and theoretical plate number.

In order to assess the proposed UPLC method as a stability-indicating procedure for the quality control of anti-TB medicines, a pure standard with concentrations of 2.8 µg/mL of EMB and 10 µg/mL of INH, PZA, and RFP was exposed to 80 °C and three fluorescent lights with a cold white light (standard illuminant D65, 6500K), in accordance with the ICH guidelines, in a climatic chamber (ICH 110L, Memmert) [3,47].

The RGB additive color model proposed by Nowak and Kóscielniak was followed to evaluate the sustainability in order to determine if it could be categorized as a green analytical method. For this task, the analytical performance (accuracy, precision, coefficient of variation, and quantification limit), safety, and eco-friendliness (liquid consumption and chemical safety), as well as the productivity and practical effectiveness (cost- and time-effectiveness), were evaluated.

### 3.5. Quality Control Application

In order to ensure that the method is capable of analyzing marketed formulations used to treat TB with accuracy and precision, three formulations were analyzed using the previously validated method. A combination of EMB, INH, PZA, and RFP commercialized in Spain (Rimstar^®^, Sandoz Farmacéutica, Madrid, Spain) was acquired from a distribution company and was used as the quality reference for the API content. Three formulations sampled in different locations in the Islamic Republic of Mauritania were also analyzed. One of them combined four APIs in the same tablet (4-FCT: EMB, INH, PZA, and RFP), the second formulation contained three APIs (3-FCT: INH, PZA, and RFP), and the last drug product only contained EMB in its composition.

An identification sheet was completed in situ for each drug product, with the relevant information related to the packaging (batch number, number of units, expiration date, manufacturer laboratory and packaging conditions), storage place (establishment type and name and storage conditions), the person responsible for the sampling, and date. After collecting the drug products, they were placed in an isothermal bag and transported to the laboratory for quality control. Upon arrival at the laboratory, the drug products were stored at 5 °C and 11% RH and analyzed before their expiration dates.

Tablets were analyzed individually after they were sampled and after six months under accelerated conditions (40 ± 2 °C/75 ± 5% RH) in a climatic chamber (ICH 110L, Memmert). This was carried out in order to evaluate the capability of the method to analyze marketed formulations, as well as its capacity for the detection of degradation products (ICH 110L, Memmert). Every solid pharmaceutical form was powdered before being analyzed, and the following procedure was carried out. In the case of the FCTs of three or four drugs, the powder was transferred to a 250 mL flask, dissolved in 75 mL of methanol, filled to 75% of the total capacity with purified water, and sonicated for 20 min. Finally, the volume was completed with purified water. For the EMB tablets, each tablet was taken and transferred to a 500 mL flask, supplemented with purified water, and stirred until complete dissolution. This procedure was repeated three times for each formulation tested. Only those drug products whose API content was between 95 and 105% of the DV were placed under accelerated conditions.

## 4. Conclusions

A UPLC method was developed, validated, and applied for the analysis of first-line anti-TB drugs for the purposes of quality control and the detection of falsified and substandard medicines. In addition, the application of DoE and MLR enabled the identification of the factors and their effects on the responses, as part of the QbD approach, for the optimization of the analytical method. This new UPLC-UV/MS method proved to be linear, accurate, and precise once it was optimized, offering a potential alternative to the current USP method, as it is able to quantify four APIs with different physicochemical characteristics using the same mobile and stationary phases in a single injection, with an appropriate retention factor for all the APIs. In addition, the inclusion of an MS detector can be useful for detecting degradation products due to its higher sensitivity. This method was applied to the quantification of APIs in commercially available formulations as part of their quality control analysis, which enabled the detection of two substandard formulations. In further studies, a complete quality control analysis of anti-TB drug products will be performed.

## Figures and Tables

**Table 1:** Factors used for the estimation of the robustness based on the central composite design and responses, retention factor (k’), and amount of API expressed as the percentage of the declared value (DV). T: temperature.

	Factors			Responses					
Run Order	T (°C)	Flow (mL/min)	Formic Acid (%)	INH		PZA		RFP	
				K’	%DV	K’	%DV	K’	%DV
15	28	0.36	0.008	1.362	110.9	5.559	109.3	13.296	113.3
5	32	0.36	0.008	1.241	115.3	4.920	110.7	13.395	107.9
8	28	0.46	0.008	1.312	89.0	5.329	87.2	15.685	92.7
17	32	0.46	0.008	1.248	90.9	4.866	86.7	15.642	98.1
16	28	0.36	0.013	1.115	115.2	5.376	109.4	13.441	111.0
7	32	0.36	0.013	1.063	125.3	4.993	107.7	13.507	113.5
19	28	0.46	0.013	1.126	89.3	5.398	87.8	15.808	93.4
4	32	0.46	0.013	1.056	88.3	4.919	85.4	15.905	75.6
2	25	0.40	0.010	1.286	101.1	5.878	96.7	14.201	98.6
12	35	0.40	0.010	1.099	99.0	4.584	97.7	14.278	99.9
11	30	0.30	0.010	1.143	137.8	5.015	131.3	11.869	129.9
9	30	0.50	0.010	1.174	96.9	5.099	80.6	16.662	89.1
3	30	0.40	0.005	1.902	98.0	6.070	98.2	16.332	93.6
14	30	0.40	0.015	0.097	102.6	5.058	97.6	14.424	84.5
1	30	0.40	0.010	1.132	104.5	4.915	101.4	14.240	100.6
6	30	0.40	0.010	1.201	104.3	5.257	98.2	14.258	101.4
10	30	0.40	0.010	1.178	102.4	5.111	98.6	14.245	100.8
13	30	0.40	0.010	1.183	104.6	5.151	100.5	14.231	100.5
18	30	0.40	0.010	1.185	103.7	5.132	100.5	14.277	103.6
20	30	0.40	0.010	1.177	105.7	5.114	101.5	14.264	100.5

**Table 2:** Evaluation of the influential factors and their effects on the responses, retention factor (k’), and percentage (%API) for each one analyzed, assuming a second-degree model. Only significant coefficients are given.

	k’			%INH		
Term	Estimate	*p* > |t|	Effect (%) ^a^	Estimate	*p* > |t|	Effect (%)
**Intercept**	1.178	<0.0001		10.429	<0.0001	
**F**				−1.302	<0.0001	12.49
**FA**	−0.173	<0.0001	14.70			
**TxT**				−0.208	0.0151	2.00
**FxF**				0.401	<0.0001	3.85
**FAxFA**	0.0762	0.0278	6.47	−0.201	0.0177	1.93
	**k’**			**%PZA**		
**Intercept**	5.112	<0.0001		10.018	<0.0001	
**T**	−0.303	<0.0001	5.93			
**F**				−1.283	<0.0001	12.81
**FAxFA**	0.133	0.0478	2.60			
	**k’**			**%RFP**		
**Intercept**	14.254	<0.0001		10.117	<0.0001	
**F**	1.278	<0.0001	8.96	−1.131	<0.0001	11.18
**FA**				−0.247	0.0453	2.44
**FxF**				0.325	0.0114	3.21
**FAxFA**	0.384	0.00463	2.69	−0.397	0.00365	3.92

^a^ The relative effect (as a percentage) was calculated by dividing the coefficient estimate by the mean of the responses, in accordance with Destandau et al. (2006) [38].

**Table 3:** Results of the method validation. API: active pharmaceutical ingredient, EMB: ethambutol, INH: isoniazid, PZA: pyrazinamide, RFP: rifampicin, CV: coefficient of variation for the analytical method (%); DL: detection limit; QL: quantification limit.

API	Linear Equation	CV (%)	Accuracy (%)	Precision (%)	DL (µg/mL)	QL (µg/mL)
**INH**	y = 16302 + 45287 × C (µg/mL); r^2^ = 0.998	2.36	100.2	0.33	0.76	2.29
**PZA**	y = 53514 × C (µg/mL); r^2^ = 0.998	1.81	99.5	0.08	0.59	1.81
**RFP**	y = 52294 × C (µg/mL); r^2^ = 0.996	3.70	97.5	0.77	1.22	3.70
**EMB**	y = 636215 + 625147 × C (µg/mL); r^2^ = 0.985	4.38	97.3	0.54	0.40	1.22

**Table 4:** Percentage of API remaining after being stored under stress conditions: temperature (80 °C) and a source of light. EMB: ethambutol, INH: isoniazid, PZA: pyrazinamide, RFP: rifampicin.

		EMB (%)		INH (%)		PZA (%)		RFP (%)	
	Condition	80 °C	Light	80 °C	Light	80 °C	Light	80 °C	Light
**Time (h)**	**0**	100.0	100.0	100.0	100.0	100.0	100.0	100.0	100.0
	**2**	88.2	98.5	90.7	97.8	100.2	100.0	0	80.8
	**4**	87.5	-	89.8	-	100.2	-	0	-
	**24**	84.2	94.2	89.5	90.6	100.6	100.5	0	24.5

**Table 5:** Mobile phase gradient for the analysis of EMB, INH, PZA, and RFP in a UPLC-UV/MS system.

Time (Min)	A (%)	B (%)	Elution
0–2.50	0	100	Isocratic
2.50–5.00	40	60	Linear gradient
5.00–6.50	40	60	Isocratic
6.50–7.00	20	80	Linear gradient
7.00–8.00	20	80	Isocratic
8.00–10.00	0	100	Linear gradient
10.00–12.00	0	100	Re-equilibration

## Data Availability

Not applicable.
